# Simple and Rapid Non-Enzymatic Procedure Allows the Isolation of Structurally Preserved Connective Tissue Micro-Fragments Enriched with SVF

**DOI:** 10.3390/cells10010036

**Published:** 2020-12-29

**Authors:** Alice Busato, Francesco De Francesco, Reetuparna Biswas, Silvia Mannucci, Giamaica Conti, Giulio Fracasso, Anita Conti, Valentina Riccio, Michele Riccio, Andrea Sbarbati

**Affiliations:** 1 Department of Neuroscience, Biomedicine and Movement, Human Anatomy and Histology Section, University of Verona, 37135 Verona, Italy; alice.busato@univr.it (A.B.); reetuparna.biswas@univr.it (R.B.); silvia.mannucci@univr.it (S.M.); giamaica.conti@univr.it (G.C.); anita.conti@univr.it (A.C.); andrea.sbarbati@univr.it (A.S.); 2 Department of Reconstructive Surgery and Hand Surgery, AOU “Ospedali Riuniti”, 60126 Ancona, Italy; francesco.defrancesco@ospedaliriuniti.marche.it (F.D.F.); michele.riccio@ospedaliriuniti.marche.it (M.R.); 3 Department of Medicine, Section of Immunology, University of Verona, 37135 Verona, Italy; giulio.fracasso@univr.it; 4 School of Biosciences and Veterinary Medicine, University of Camerino, 62024 Matelica, Italy; valy.riccio91@gmail.com; 5 Research and Training Center in Regenerative Surgery, Accademia del Lipofilling, 61025 Montelabbate, Italy

**Keywords:** stromal vascular fraction, adipose tissue, non-enzymatically extraction, mesenchymal stem cells, micro-fragments, regenerative medicine

## Abstract

The stromal vascular fraction (SVF) consists of a heterogeneous population of stem and stromal cells, generally obtained from adipose tissue by enzymatic digestion. For human cell-based therapies, mechanical process methods to obtain SVF represent an advantageous approach because they have fewer regulatory restrictions for their clinical use. The aim of this study was to characterize a novel commercial system for obtaining SVF from adipose tissue by a mechanical approach without substantial manipulations. Lipoaspirate samples collected from 27 informed patients were processed by a simple and fast mechanical system (by means of Hy-Tissue SVF). The Hy-Tissue SVF product contained a free cell fraction and micro-fragments of stromal connective tissue. The enzymatic digestion of the micro-fragments increased the yield of free cells (3.2 times) and CFU-F (2.4 times). Additionally, 10% of free cells from SVF were positive for CD34+, suggesting the presence of endothelial cells, pericytes, and potential adipose-derived stem cells (ADSC). Moreover, the SVF cells were able to proliferate and differentiate in vitro toward adipocytes, osteocytes, and chondrocytes. The immunophenotypic analysis of expanded cells showed positivity for typical mesenchymal stem cell markers. The Hy-Tissue SVF system allows the isolation of stromal vascular fraction, making this product of potential interest in regenerative medicine.

## 1. Introduction

Biological therapies have become popular in the last few decades since they represent an alternative to the classical pharmacologic or surgical approaches to treat acute and degenerative chronic injuries. In this sense, adipose tissue has been postulated as an important biological product for the treatment of chronic and acute degenerative conditions since it is a source rich in adult multipotent stem cells [1].

Stromal Vascular Fraction (SVF) represents a freshly isolated heterogeneous cell mixture present in the adipose tissue. It comprises smooth muscle cells, preadipocytes, endothelial cells (ECs), and several multipotent adult stem cells, such as endothelial and hematopoietic progenitor cells (EPC), pericytes, and adipose-derived stem cells (ADSC), among others [2,3]. The higher mesenchymal stem cell (MSC) concentration in the native adipose tissue has led researchers and clinicians to focus from the bone marrow source to the adipose tissue. For example, the frequency of MSC in bone marrow is 0.01–0.001% cells [4], while adipose tissue contains 500 times more MSC per volume of fat as compared to the volume of bone marrow [2]. Moreover, ADSC has been proven to be useful for modifying inadequate healing responses that lead to the degeneration of a tissue and its improper remodeling, such as chronic inflammation [5], fibrosis [6], and hypermetabolic responses [7]. Thus, SVF is considered of singular importance due to its potential in regenerative medicine, particularly for osteoarthritis treatment [8,9,10]. SVF can be obtained from adipose tissue by enzymatic or non-enzymatic processes [11]. Enzymatic digestion of adipose tissue is the most common isolation technique and is based on the utilization of proteases, such as collagenases, which digest the tissue matrix [12]. This procedure results in an SVF cell product with a high cellular yield with a high frequency of progenitor cells. However, it is time-consuming (requires a 30–45 min incubation step with a controlled temperature at 37 °C) and the cells obtained are considered substantially manipulated, which makes the product to be classified as an advanced-therapy medicinal product (ATMP) by regulatory authorities in Europe and USA (Regulation (EC) No 1394/2007; 21 CFR 1271.10, respectively). Alternatively, non-enzymatic methods (mechanical methods) use physical forces to disrupt the tissue and obtain cells from the adipose stroma [13]. This type of procedure has the advantage that it is not considered as a substantial manipulation, and, on the other hand, it is faster and simpler than the enzymatic method.

The aim of this work is to characterize a novel commercial system (Hy-Tissue SVF) for obtaining SVF by mechanical disruption of adipose tissue without substantial manipulations.

## 2. Materials and Methods

### 2.1. Adipose Tissue Collection

The adipose tissue was collected from 27 women undergoing liposuction for aesthetic purposes, aged between 41 and 69 years. Informed consent was taken prior to the collection of adipose tissue in accordance with the ethical guidelines established by the review committee for human studies of AOU “Ospedali Riuniti”, Ancona, Italy (Micro-adipose graft_01, 18 May 2017). In brief, Klein tumescence solution (2% Lidocaine solution: 0.08% *w/v*; Adrenaline 1mg/mL solution: 0.1% *v/v* in 0.9% saline) was injected and after 10 min liposuction started. A cannula of 11 G, 6 holes, and 20 mL Vac-Lock syringe provided with the kit was employed to lipoaspirate between 30 mL of fat from each donor’s abdominal area. The fat was transported in an adiabatic container to the laboratory and processed within 18 h from harvest.

### 2.2. Procedure for SVF Production

Each sample of adipose tissue (about 30 mL) was decanted to remove excess oil and divided into 2 portions. The first portion of the lipoaspirate sample was processed by different trained technicians with the Hy-Tissue SVF kit (Fidia Farmaceutici, Abano Terme, Italy) through a mechanic disaggregation process. The kit provided a sterile, single-use, tissue collection double bag with an inner filter bag of 120 µm mesh. A volume of lipoaspirate (25–30 mL) was transferred into the inner bag by the upper port. Placing the bag vertically, the Klein solution containing part of blood cells was recovered in the lower part of the processing bag and removed while the adipose tissue remained in the inner filter bag. An equal volume of PBS solution equal to Klein solution removed was introduced into the processing bag through the upper port, and the fat was processed according to the instruction for use. Briefly, fat tissue was massaged for 5 min and disaggregated by using a small plastic rod and enforced to pass through the filter bag by manual massaging. The disaggregated tissue was collected with a syringe using the lower valve port of the outer bag and centrifuged at 400 G for 10 min at room temperature, followed by resuspension in 1 mL of Dulbecco Minimum Essential Medium (DMEM) complete culture medium (Sigma-Aldrich, Milan, Italy) with 10% of Fetal Bovine Serum (Sigma-Aldrich, Milan, Italy)), 0.5% of amphotericin B (GIBCO Life Technology, Monza, Italy), and 1% of a mixture of penicillin/streptomycin 1:1 (GIBCO Life Technology, Monza, Italy) to count the number of cells inside. The product obtained by this method was named as “SVF”.

### 2.3. Enzymatic Digestion of the Fat

The 2nd portion of lipoaspirate (5 mL) was processed using an enzymatic method as reported in Dai Pre et al., 2020. Briefly, fat samples were digested with collagenase type I at the concertation of 1 mg/mL (GIBCO Life Technology, Monza, Italy) resuspended in Balanced Salt Solution of Hank (HBSS, GIBCO Life Technology, Monza, Italy) and bovine serum albumin (BSA, 2%, GIBCO Life Technology, Monza, Italy) at 37 °C for 45 min. Complete culture medium was added to neutralize the enzyme action, and the sample was centrifuged at 400 G for 10 min. After centrifugation, the pellet was incubated with 2 mL of lysis buffer for 10 min. The cell suspension was then centrifuged and resuspended with 2 mL of complete culture medium. The product obtained by this method was named as “FAT-ED”.

### 2.4. Enzymatic Digestion of the SVF

Adipose tissue (25 mL) of N = 5 patients was subjected to Hy-tissue SVF treatment using the described protocol. An aliquot of each SVF product was stored for proliferation capacity and CFU-F analysis of the free cells portion while the rest was centrifuged at 400 G for 10 min. The pellet obtained was resuspended in 1 mL of 1 mg/mL of collagenase type I (GIBCO Life Technology, Monza, Italy) solution in the HBSS (GIBCO Life Technology, Monza, Italy) and 2% of BSA (GIBCO Life Technology, Monza, Italy) for 20 min at 37 °C inside a shaking incubator, then the complete medium was added (1:1 *v*/*v*) to block enzymatic digestion, and the digested material was centrifuged (400 G, 10 min). The product obtained by this method was named as “SVF-ED”.

### 2.5. Cell Counting and Yield

Free cells were counted using Trypan Blue exclusion assay using a Burker chamber manually and with CytoSMART counter (Automated Image-Based Cell Counter, version 1.5.0.16380, CytoSMART Technologies B.V, Eindhoven, The Netherlands). Cell or CFU-F yield was calculated considering the total amount of free cells of SVF, SVF-ED, or FAT-ED divided by the volume of fat processed after discarding the Klein solution.

### 2.6. Cell Proliferation Capacity

To determine the cell proliferation capacity of the free cells contained in either SVF, SVF-ED, or FAT-ED, 2 × 10^5^ cells of each product were plated on a 25 cm^2^ T-flask and incubated at 37 °C with 5% CO_2_. Three days after the cell extraction, the complete medium was changed and then every 48 h until 80% confluence. The days the culture required to reach confluence (passage 1) were used for determining the proliferation capacity. Moreover, in order to estimate the time required by cells to duplicate their number, population doubling time assays were performed. Four days after the seeding, 5 × 10^4^ cells from “SVF,” “SVF-ED,” and “FAT-ED” were plated in T-25 Flasks (in triplicates) and cultured with 5 mL of complete media and incubated at 37 °C, 5% CO_2_, for 24, 72, and 96 h. At each time point, cells were washed with PBS 1X, detached with trypsin/EDTA, resuspended in 1 mL of media, and counted using a CytoSMART counter (Automated Image-Based Cell Counter, version 1.5.0.16380, CytoSMART Technologies B.V, Eindhoven, The Netherlands). The population doubling time (pdt) was calculated using the following equation: pdt = [t(h)*log2]/log (Nf/Ni) (as reported in Martinello T. et al., 2010), where Ni and Nf are initial and final cell numbers, respectively.

### 2.7. Colony Forming Unit Assay

Free cells from SVF, SVF-ED, or FAT-ED were seeded in a 6-well plate at 1000 and 5000 cells/well in triplicate. Cells were cultured for 14 days. Toluidine Blue (Sigma-Aldrich, Milan, Italy) staining was performed to count the colony, and only clusters of at least 50 fibroblast-like cells were considered. The frequency of CFU-F within SVF, SVF-ED, or FAT-ED was expressed as a percentage of seeded cells.

### 2.8. Optical Microscopy

SVF pellets (N = 8) obtained after Hy-Tissue SVF treatment were analyzed by the whole-mount method (Larsen PL 1989) and paraffin included. Briefly, N = 4 pellets were washed with saline, stained with Hematoxylin and Eosin (Bio-Optica, Milan, Italy), and placed on the slide without dissection with the whole-mount method. Other pellets (N = 4) were washed with PBS and fixed with 10% formalin (Sigma-Aldrich, Milan, Italy) for 4 h, dehydrated until xylene, and included in paraffin. Sections of 5 µm were cut with a microtome into sections. The sections were then dried at 37 °C for 24 h and stained with Hematoxylin and Eosin (Bio-Optica, Milan, Italy). All slides were examined under an Olympus BX51 microscope (Olympus, Tokyo, Japan) equipped with a digital camera (DKY-F58 CCD JVC, Yokohama, Japan). The digital images were analyzed with Image-ProPlus 7.0 software (Media Cybernetics, Silver Spring, MD, USA).

### 2.9. SEM Analysis

For scanning electron microscopy analysis (SEM), SVF pellets (N = 4) were fixed in 2% glutaraldehyde (Sigma-Aldrich, Milan, Italy for 4 h, postfixed in 1% osmium tetroxide (Sigma-Aldrich, Milan, Italy) for 1 h, and dehydrated in graduated acetone concentrations (Sigma-Aldrich, Milan, Italy). The samples were treated with a critical-point dryer (CPD 030, Balzers Vaduz, Liechtenstein), mounted on metal samples, and coated with gold (MED 010 Balzers). SEM images were acquired with XL30 ESEM (FEI-Philips, Eindhoven, The Netherlands).

### 2.10. Immunophenotyping

Free cells from SVF and SVF-ED products, as well as the subsequent subculture cells (named as passage 1–P1), were characterized by flow cytometry. For this purpose, SVF and FAT-ED products were passed through a 45 µm cell-strainer to remove micro-fragments and cell clumps. Then, 0.2 × 10^5^ cells were washed with 1 mL in PBS (1X Cells were incubated with conjugated antibodies for 30 min. After incubation, the pellets were centrifuged (7000 rpm, 5 min) and resuspended in 300 µL of PBS (1X). The antibodies used were: CD90 APC conjugate (1:5 dilution), CD105.5 PerCP-Cyt5.5 conjugate (1:20 dilution), CD73 BV421 conjugate (1:20 dilution); CD44 BV785 conjugate (1:20 dilution), CD34 PE conjugate (1:5 dilution), CD29 FITC conjugate (1:20 dilution), CD45 FITC conjugate (1:20 dilution), CD146 APC conjugate (1:20 dilution), CD68 FITC conjugate (1:20 dilution), CD116 FITC conjugate (1:20 dilution). For cell viability, Propidium Iodide was used. All antibodies were purchased from BD Biosciences (Becton Dickinson Italy S.P.A., Milano, Italy). Immunophenotyping was performed through a chant II FACS (BD, Becton Dickinson, Milano, Italy). Moreover, we performed a white blood cell differential (WBC diff) count on the Advia 120 automated hematology analyzer (Bayer Diagnostics, Berkshire, Newbury, UK). The WBC were divided into four main cell subpopulations, corresponding to neutrophils (NEUT), lymphocytes (LYMPH), monocytes (MONO), eosinophils (EO). Basophils (BASO) were counted in a separate channel.

### 2.11. In Vitro Differentiation Assays

The differentiation potential was evaluated in vitro for SVF and SVF-ED (control). Differentiation was carried out employing expanded cultured cells from passage 4. For adipogenic differentiation, 7000 cells were seeded on a 6-well plate, incubated, and after 24 h, the media was replaced with adipogenic media (Sigma-Aldrich, Milan, Italy) and incubated for 16 days. Then, cells were fixed with 4% paraformaldehyde (PFA) for 30 min at 4 °C, washed twice with PBS, and stained with Oil Red O (Bio-Optica, Milan, Italy) for 20 min and Hematoxylin (Bio-Optica, Milan, Italy) for 1 min. For chondrogenic differentiation, 1 × 10^6^ cells resuspended in 5 µL of complete media were seeded in a 24-well plate, and after 2 h, the chondrogenic media was added (StemPro chondrogenic differentiation Kit -GIBCO Life Technology, Monza, Italy). After 14 days of incubation, changing of the media every 3 days, cells were fixed with 4% PFA for 30 min at 4 °C, washed twice with PBS, and stained with Alcian Blue 8GX (Sigma-Aldrich, Milan, Italy) to detect mucopolysaccharide extracellular matrix. For osteogenic differentiation, 5000 cells were seeded on a 12-well plate with complete media, and after 24 h, the media was changed with osteogenic media (StemPro osteogenesis differentiation Kit–GIBCO Life Technology, Monza, Italy). After 21 days, cells were fixed with 4% PFA for 30 min at 4 °C, washed twice with PBS, and incubated with 0.2% Alizarin Red S (Sigma-Aldrich) for 5 min and Hematoxylin (Bio-Optica, Milan, Italy) for 1 min. Images were obtained using optical microscopy.

### 2.12. Statistical Analysis

We have reported data as mean ± standard deviation (SD). Statistical analysis was performed with a Mann–Whitney test. An alpha value of *p* < 0.05 was regarded as statistically significant. All statistical analyses were performed using GraphPad Prism version 7.00 for Windows, GraphPad Software, La Jolla, CA, USA.

## 3. Results

**Figure 1:**
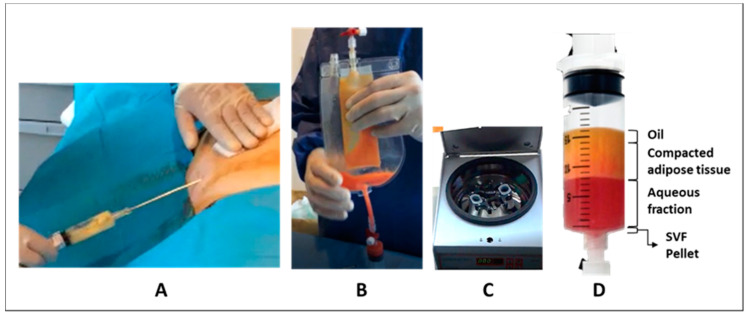
Diagram of the Hy-Tissue stromal vascular fraction (SVF) process. (**A**) Lipoaspiration; (**B**) mechanic disaggregation of adipose tissue using the double bag; (**C**) centrifugation, (**D**) phase separation: From up to down, it is possible to distinguish the following layers: Oil, condensed fat, aqueous fraction, and a bottom pellet with the SVF product.

We have investigated the composition of the SVF obtained with the Hy-Tissue SVF kit. For this purpose, 22 lipoaspirates were processed following the protocol recommended by the manufacturer. Once the adipose tissue was filtered (and disrupted) by the nylon membrane of 120 um, the centrifugation step separated the disrupted tissue into three different layers, based on the water or lipid content. The upper phase consisted of a liquid oily phase resulted from the release of stored oil from mature adipocytes. Then, there is a phase of condensed adipose tissue (Coleman-like), which still have adipocytes allowing it to float in an aqueous solution, and finally a non-floating pellet, which contains the SVF (Figure 1).

### 3.1. Microscopical Analysis of the SVF

**Figure 2:**
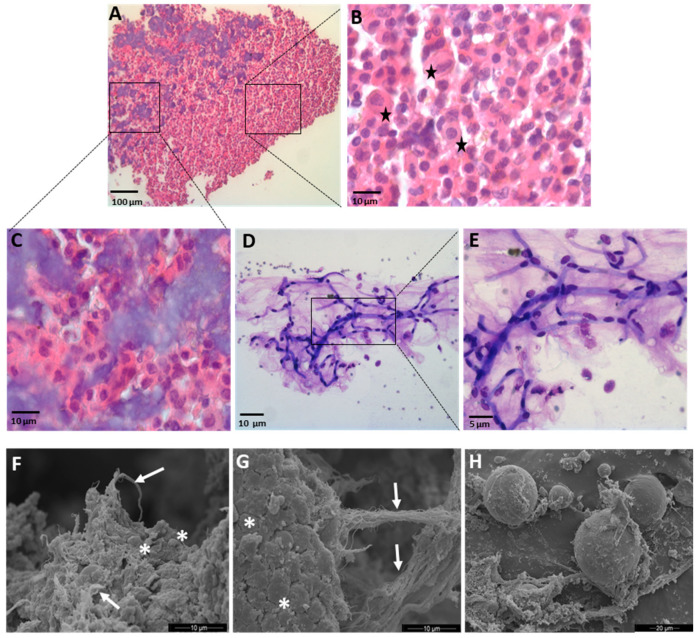
Morphological and ultrastructural analysis of Hy-Tissue SVF product. (**A**–**C**) Light microscopy of a single-layer section of pellet stained with Hematoxylin and Eosin. Black stars in (**B**) highlight cell heterogeneity. The squares indicate the location of the higher magnification image in (**B**,**C**). (Scale bar: (**A**) 100 μm, (**B**,**C**) 10 μm). (**D**,**E**) Hematoxylin and Eosin staining of connective tissue micro-fragments obtained from Hy-tissue SVF. Preserved microvascular elements appear as tubular structures (stained in deep purple) containing endothelial and perivascular cells. The rectangle indicates the location of the higher magnification image in (**E**) (scale bar: (**D**): 10 μm; (**E**): 5 μm). (**F**–**H**) SEM images of connective tissue micro-fragments. Arrows: collagen fibers; asterisks: adherent cells. SEM allows to identify also small adipocytes in Hy-Tissue SVF product (**H**) (Scale bar: (**F**,**G**): 10 μm; (**H**) 20 μm).

Microscopical analysis of the sediment obtained with the mechanical dissociation process of the fat (SVF) revealed the presence of a cells pellet (Figure 2A) composed of free cells (Figure 2B) and micro-fragments (Figure 2C) of stromal connective tissue. Among the free cells, a heterogeneous cell population was clearly observed), and stars in Figure 2B highlight the differences in size and shape of the nuclei and cytoplasm (see stars in Figure 2B). The micro-fragments appeared as elongated cylinders with an average diameter of about 30–70 µm of variable length with a fibrinoid assembly with tubular structures (stained in deep purple), which represents microvascular elements consistent in capillaries of variable length containing endothelial and perivascular cells (Figure 2D,E). The evidences of optical microscopy were supported by SEM images (Figure 2F,H). The micro-fragments were composed of dense connective tissue with the presence of collagen fibers structured as isolated fibrils (Figure 2F, arrow) or coarse bands (Figure 2G, arrow) containing adherent cells (Figure 2F,G asterisk). Moreover, SEM images show the presence of adipocytes (Figure 2H) that appear of small size (10–20 μm), attributable to adipocytes that have lost their lipid content or are not completely differentiated.

### 3.2. In Vitro Characterization of the SVF

The SVF products obtained by the mechanical disaggregation process of the Hy-Tissue SVF kit were analyzed in terms of cell yield, product quality, viability, proliferation capacity, immunophenotyping, and differentiation potential.

**Figure 3:**
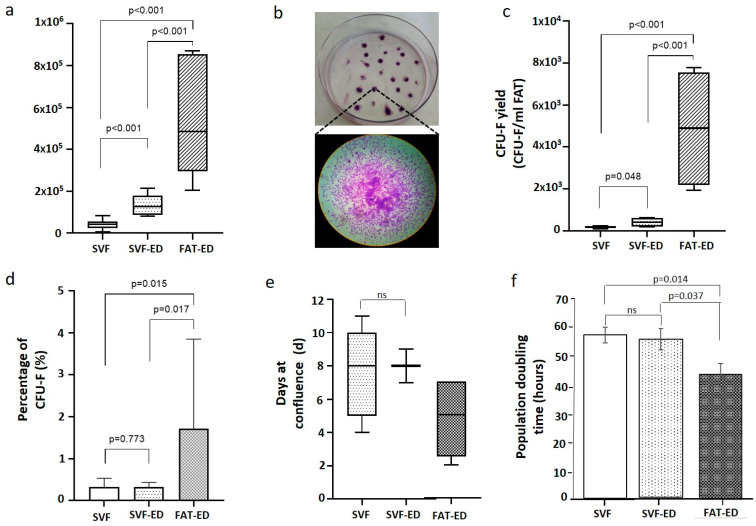
(**a**) Nucleated cells of the SVF obtained after treatment with Hy-Tissue SVF and the SVF after enzyme digestion of the micro-fragments (SVF-ED). Fat tissue enzymatically digested (FAT-ED) was used as a control.; (**b**) representative T –flask of the CFU-F assay and an amplified image of a CFU-F observed with a light microscope after Toluidine Blue staining (4× magnification). (**c**) CFU-F yields of the SVF obtained after treatment with Hy-Tissue SVF and the SVF after enzyme digestion of the micro-fragments (SVF-ED). Fat tissue enzymatically digested (FAT-ED) was used as control; (**d**) percentage of CFU-F contained in SVF, SVF-ED, or FAT-ED. Results are presented as the mean and error bars one standard deviation; (**e**) proliferation capacity of cells contained in SVF, SVF-ED, or FAT-ED in T25 flasks. Box and whisker plots represent the median, the lower and upper quartile, and the minimum and maximum; (**f**) population doubling time of SVF, SVF-ED and FAT-ED. The results are shown as the mean and error bars represents standard deviation.

The cell yield of free nucleated cells of the SVF product was 4.1 × 10^4^ ± 2.0 × 10^4^ cells/mL Fat (Figure 3a). When the SVF pellet (containing free cells and adipose tissue micro-fragments) was submitted to enzymatic digestion, the product obtained (SVF-ED) increased 3.2 times the yield of free nucleated cells (1.3 × 10^5^ ± 4.7 × 10^4^ cells/mL FAT; Figure 3a). To evaluate the ability to form colonies of Hy-Tissue SVF product, colony-forming unit-fibroblast (CFU-F) assays were performed. CFU-F assay revealed that CFU-F yield of SVF was 178 ± 49 CFU-F/mL FAT and after enzymatic digestion of the micro-fragments of the SVF increased over 2.4 times (424 ± 181 CFU/mL FAT, Figure 3c). The relative proportion of CFU-F in the SVF (0.3 ± 0.2%) did not increase significantly after the enzyme digestion of the micro-fragments (Figure 3d), suggesting that the micro-fragments were composed not only of cells with adhesion potential but also of other non-adherent cell types. Free-cells yield and free-cells CFU-F of SVF were compared to fat tissue digested enzymatically (FAT-ED), used as a control. FAT-ED yielded 12-times more nucleated cells (5 × 10^5^ ± 2 × 10^5^ cells/mL FAT) and 27-times more CFU-F (4877 ± 2477 CFU-F/mL FAT) compared to the SVF component based on the free cells fraction obtained by mechanical procedures. Additionally, the frequency of CFU-F of the fat tissue treated with enzymes (FAT-ED) was 5.6 times higher than the SVF product (FAT-ED: 1.7 ± 1.9%; SVF: 0.3 ± 0.2%; SVF-ED: 0.3% ± 0.1). Finally, to analyze the capacity of the proliferation of adherent cells in the SVF product, cells from SVF, SVF-ED, or FAT-ED (control) were seeded in T-flasks until confluence (Figure 3e). SVF and SVF-ED cells did not show statistically significant differences to reach confluence (8 ± 2 days; 8 ± 1 days, respectively; *p* > 0.05). Fat-ED (control) required less time to reach confluence (5 ± 2 days), probably due to the higher frequency of adherent cell content. Proliferation capacity results were confirmed with a population doubling time assay (Figure 3f). Histograms show that the doubling time of SVF and SVF-ED cells was comparable (59.35 ± 2.8 and 57.38 ± 3.9 h, respectively), while cells from FAT-ED required less time to duplicate their number (45.31 ± 3.95 h).

### 3.3. Immunophenotypic Analysis

**Figure 4:**
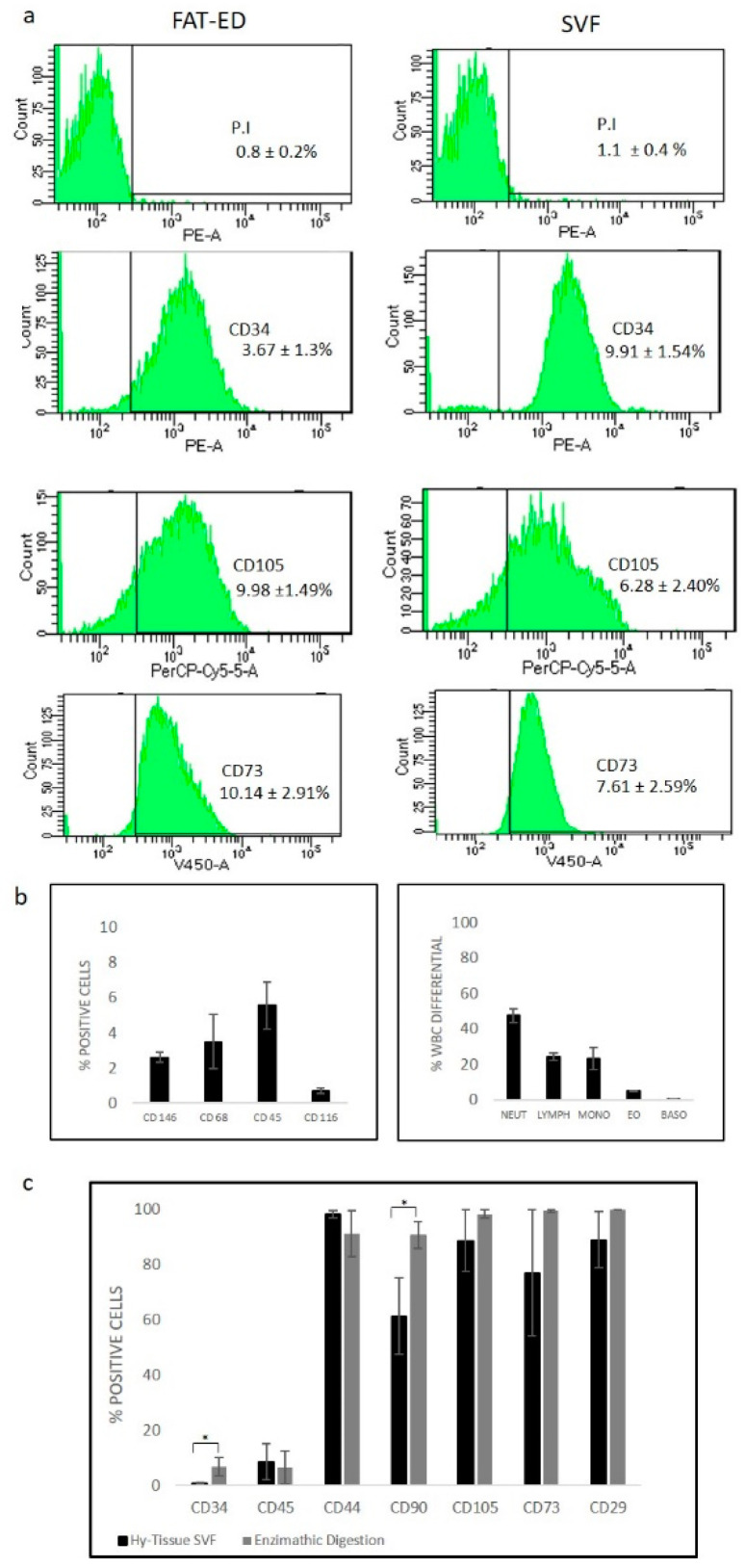
Expression of surface markers detected by flow cytometric analysis of free cells from SVF or FAT-ED (**a**). The percentage of positive cells for each marker was calculated after subtraction of the non-specific fluorescence obtained with the control (unmarked). Data show a representative set of dot-plot from one individual in each isolation protocol. (**b**) Percentage of positive cells to CD markers to characterize cell subpopulations in SVF (left), White Blood Cells differential (WBC diff) to characterized the neutrophils (NEUT), lymphocytes (LYMPH), monocytes (MONO), eosinophils (EO), and basophils (BASO) subpopulation in SVF (percentage of positive cells). (**c**) Percentage of positive cells to CD markers (as an average of the samples) after in vitro cell expansion for SVF or FAT-ED (control). Results are presented as the mean and error bars one standard deviation; significant statistical differences are indicated with “*”, *p*-value < 0.05.

To determine the stromal vascular fraction composition obtained with Hy-Tissue SVF, we used flow cytometry to evaluate the proportions of CD34+ (endothelial cells, pericytes, and potential ADSC) in the free cell fraction. Cells from a scatter plot (FSC/SSC) were analyzed for viability, and dead cells were excluded. The proportion of CD34+ cells in SVF fraction was 9.9 ± 1.5% (Figure 4a) compared to the cells obtained after enzyme digestion of the fat (3.7 ± 1.3%). Moreover, the frequency of CD73 and C105 positive cells (mesenchymal stem cells marker) were analyzed (Figure 4a), and the percentage of expression was 7.61 ± 2.59% and 6.28 ± 2.40% for CD73 and CD105, respectively (Figure 4a), not thus far from the percentage of expression obtained for the same antibody with ED (control). In order to deeper characterize the cellular composition of SVF obtained with Hy-Tissue SVF kit, flow cytometry was performed, labeling SVF cells with CD146, CD 116, CD68 and CD45. The percentage of maker expression was reported in Figure 4b (left). The proportion of CD146, CD116, CD68, and CD45 cells in SVF fraction was 2.6 ± 0.2%, 0.7 ± 0.1%, 3.5 ± 1.5%, and 5.5 ± 1.34%, respectively. Among the WBC fraction, four main cell subpopulations were determined and the percentage of expression for neutrophils, lymphocytes, monocytes, eosinophils, and basophils was reported in Figure 4b (right).

Figure 4c shows the immunophenotypic analysis of surface marker expression profiles of MSC obtained after the cellular expansion of the adherent cells obtained from SVF or enzymatic digestion of the FAT (FAT-ED) were similar. As expected, most of the cells have expression low for CD45, as well as CD34 and positive for MSC-associated markers such as CD105, CD90, CD73, CD29, and CD44 (Figure 4c).

### 3.4. Analysis of Multipotency of SVF Product

**Figure 5:**
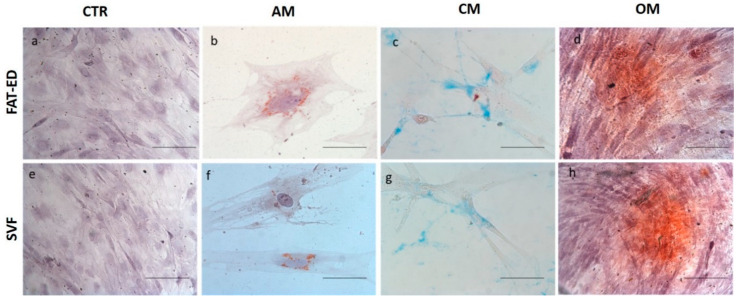
Multilineage differentiation of passage 4 of SVF or FAT-ED adipose-derived stromal cells isolated by each method. AM: Adipogenic medium; CM: Chondrogenic medium; OM: Osteogenic medium. (**a**–**h**): Optical microscopy images of cells induced or not (control; **a**,**e**) with differentiation medium (scale bar 100 μm); (**b**,**f**) adipogenesis was indicated by the accumulation of neutral lipid vacuoles that stain with Oil Red O; (**c**,**g**) chondrogenesis was indicated by the deposition of sulfated proteoglycan-rich matrix that stained with Alcian Blue; (**d**,**e**) osteogenesis was indicated by Alzarin red S staining of extracellular matrix calcification.

The multilineage differentiation potential of the SVF obtained with Hy-Tissue SVF was evaluated by testing the ability of adherent cells obtained in the SVF to differentiate toward adipocytes, chondrocytes, and osteocytes using the expanded cells from FAT-ED as a control. Peripheral blood contaminants and other non-adherent stromal cells of the SVF were removed by replating until passage 4, and only adherent cells with fibroblastoid morphology remained in the flask. The results of the multipotency of SVF product are shown in Figure 5. Adherent cells obtained with Hy-Tissue SVF are able to differentiate into the three mesodermal lineages as the cells enzymatically extracted (FAT-ED). Oil Red O, Alizarin Red, and Alcian Blue staining were positive in comparison with the control for adipogenic, osteogenic, and chondrogenic differentiation, respectively.

## 4. Discussion

The technique evaluated was simple and provided SVF in the form of free cells and micro-fragments within a short time (15–20 min), without expansion and/or enzymatic treatment.

The technique reduced the size of the cluster inside the adipose tissue (previously fragmented by the lipoaspiration procedure) by gently forcing the tissue to pass through a sieve of 120 µm (filtered microfat) and a subsequent centrifugation step that allowed to break most of the mature adipocytes for finally obtaining a pellet with free cells (blood and stroma origin) and micro-fragments of adipose tissue without mature adipocytes, which were broken by the mechanical disaggregation and the centrifugal forces.

A relevant aspect of this study was the detection of micro-fragments in the SVF pellet. Indeed, the morphological characterization of Hy-Tissue SVF product revealed the presence of the micro-fragments of connective tissue that preserved part of the original architecture of adipose tissue with multiple cell types but without mature adipocytes. In fact, only a few small adipocytes (probably pre-adipocytes) were observed by SEM microscopy. We also found the presence of preserved micro-vessels within the micro-fragments with the perivascular niche apparently left unaltered. We believe that the presence of the perivascular cells could represent an important feature of the Hy-Tissue SVF product since perivascular cells behave as MSCs in vivo [14]. Actually, micrografts of fat could have a superior therapeutic potential for tissue repair and regeneration compared to enzymatically extracted ADSC by the enhanced secretory activity of growth factors and cytokines of the cells [15]. Moreover, ADSC in their own niche prolong the long-term survival of their ADSC content and induce a long-lasting anti-inflammatory activity [16]. Other authors suggest that the presence of endothelial cells and ADSC in the SVF could be advantageous since it can induce proliferative, proangiogenic, and vasculogenic effects through both paracrine mechanisms and/or cell-cell contact [17,18,19,20,21]. Additionally, the fat of the extracellular matrix plays an important role in the effectiveness of some indications, such as healing of chronic and acute wounds promoting the healing by the differentiation of multipotent cells into fibroblasts [22,23]. Moreover, the employ of autologous microfragmented adipose tissue with SVF in patients with knee OA increases the levels of GAG in cartilage, reducing pain and improving movement abilities [17].

In order to control variables that could affect the cell yield, for women, only a narrow range of ages were included in our study. Besides, lipoaspirations were done by the same physician and using the same technique. It is known that intrinsic factors such as anatomic donor site, harvesting technique, and age can affect the cell yield of the SVF product [24,25,26]. In fact, a normal lipoaspirate can yield between 1 × 10^5^ up to 1 × 10^6^ nucleated cells/mL processed lipoaspirate [27,28,29,30,31,32], which represents a high variability, probably due to the nature and complexity of adipose tissue and intrinsic factors. Besides, the yield of ADSC from an enzymatic digestion process can be between 1–5% of the total nucleated cells, depending on the donor site [33]. However, even controlling these variables, we found a high variability between fat samples in terms of cell content and yield.

In our study, we recovered up to 25% of the total stromal vascular fraction (131.200 cell/mL FAT) when both fractions (free cells and micro-fragments) were considered, compared to the stromal vascular fraction obtained by enzymatic digestion, suggesting that the process of the Hy-Tissue SVF system was highly efficient for separating nucleated stromal cells. Other mechanical methods produce similar or less nucleated cells yield compared to Hy-Tissue SVF. For instance, Markarian et al. examined a non-enzymatic procedure with centrifugation that recovered near 10,000 nucleated cells/mL of lipoaspirate [34]. Baptista et al. reported a similar proportion to our results of adherent cells obtained by enzymatic digestion vs. the mechanical manual method evaluated (10:1) [35]. Shah et al. evaluated a similar method using PBS instead of RBC lysis buffer and reported an average of 25,000 ADSC/mL of processed lipoaspirate [36]. Raposio et al. isolated about 125,000 nucleated cells per ml of processed lipoaspirate, and about 5% of these cells were probably progenitor cells detected by flux cytometry but not confirmed by CFU-F, which in our opinion is a more reliable technique for identifying multipotent cells [37]. Due to the high variability in terms of methods and results [38], we considered that the most reliable and best comparator were the results obtained for the FAT-ED of each lipoaspirate rather than the results of other described adipose-processing platforms. In our study, the mean CFU-F yield obtained in the SVF was 10 times lower compared to CFU-F yield obtained by the enzymatic digestion of fat tissue, which is a reasonable yield value considering that mechanical forces are not efficient for releasing cells attached to their natural matrix niche [39] and that cells can be retained in the phase of condensed adipose tissue (Coleman-like) formed after centrifugation. However, this result does not necessarily imply an inferior potential of mechanical SVF for therapeutic purposes. For instance, the doses of expanded ADSCs used in clinical trials are much higher than any system producing SVF [40,41]. However, both products (ADSCs and SVF) have shown similar profile improvements in terms of pain, function, stiffness, and quality of life in comparative clinical trials, suggesting that the ADSCs dose is not the most relevant variable for obtaining a therapeutic effect [10,42]. In vitro, ADSCs obtained by mechanical dissociation proved to have immunosuppressive properties like those obtained by enzymatic digestion [43]. In a randomized controlled clinical trial, SVF obtained with the Hy-Tissue SVF kit showed to be an effective treatment for recalcitrant Achilles tendinopathy [44]. The previous study also demonstrated the immunomodulatory potential of SVF in co-culture systems with activated lymphocytes by the reduction of secretion of IL-6.

It is well known that CD34 is a marker of the stromal cell-containing population [3]. In this sense, the detection of CD34+ expression in the free cell fraction of SVF, together with CD73 and CD105, the latter two being classical MSC markers [45], suggests that the free cells of this fraction contain not only nucleated blood cells of hemopoietic origin but also stromal cells. Moreover, in the free cells fraction of SVF has been found cells positive for the endothelial marker (CD146). Endothelial cells, together with stromal cells, are crucial for the long-term viability and proliferation of MSC [18,19]. Next studies should be devoted to characterize the SVF after enzymatic digestion, which probably would have provided a more reliable profile of the total type of cells of the Hy-Tissue SVF product, such as pericytes fraction, which was very low in our analysis.

The main limitations of our study were the small patient population and the no discrimination of peripheral blood cells from the cell’s free fraction. However, considering that the cell-free fraction was around 30% of the total nucleated cell content of SVF, and that near 10% were positive for CD34, we estimated that the blood contamination was less than 25% of the total cells, which in our opinion is not relevant considering the high variability of the adipose tissue. Finally, although this study characterized Hy-Tissue SVF product at a morphological, structural, and cellular level, further application studies aimed to investigate the functionality of the product are necessary.

## 5. Conclusions

In this study, we demonstrated that this innovative device allows the isolation of stromal vascular fraction in the form of free cells and micro-fragments of connective tissue containing stromal cells and extracellular matrix, making this product of potential interest in regenerative medicine.

## Data Availability

The data used to support the findings of this study are included within the article.
